# Programme guidelines for promoting good oral health for children in Nigeria: a position paper

**DOI:** 10.1186/1472-6831-14-128

**Published:** 2014-10-21

**Authors:** Morenike O Folayan, Abiola A Adeniyi, Nneka M Chukwumah, Nneka Onyejaka, Ayodeji O Esan, Oyinkan O Sofola, Omolola O Orenuga

**Affiliations:** Department of Child Dental Health, Obafemi Awolowo University, Ile-Ife, Nigeria; Department of Child Dental Health, Obafemi Awolowo University Teaching Hospitals Complex, Ile-Ife, Nigeria; Paediatric Dentistry Working Group, Ile-Ife, Nigeria; Department of Preventive Dentistry Lagos State University College of Medicine, Lagos, Nigeria; Department of Preventive and Community Dentistry, University of Lagos, Lagos, Nigeria; Department of Preventive and Community Dentistry, Obafemi Awolowo University Teaching Hospitals Complex, Ile-Ife, Nigeria; Department of Child Dental Health, University of Lagos, Lagos, Nigeria

**Keywords:** Nigeria, Children, Caries, Campaign, Evidence, Oral health promotion

## Abstract

**Background:**

The objective of this paper is to draw attention to the oral health needs of children in Nigeria, and promote the use of appropriate interventions for disease prevention in the population. It also evaluates the value of the ongoing twice-daily tooth brushing campaign, which focuses on promoting good periodontal health and its relevance for children in Nigeria.

**Discussion:**

The main oral health burden for children in Nigeria is untreated dental caries, attributable to low utilization of oral health facilities. While there is a strong association between oral hygiene status and caries occurrence, no research had established an association between frequency of tooth brushing and caries in children in Nigeria. Prevalence of caries and gingivitis is low, despite the fact that a majority of children brush once a day and most of them have fair oral hygiene. Campaigns that promote twice daily brushing to prevent chronic periodontitis in children are not driven by evidences supporting the local epidemic, and therefore cannot be considered as efficient use of the limited resources available.

**Summary:**

Existing evidences show that the main oral health need of children in Nigeria is the management of untreated caries. Promoting the treatment of caries should be the primary focus of oral health programmes for children in Nigeria, as this would reduce further risks of developing new carious lesions. Public health campaigns should focus efforts at creating demand for oral health care services, for both preventive and curative purposes.

## Background

The introduction of the National Oral Health Policy has fostered renewed interest in oral health in Nigeria. One group that has a potential to greatly benefit from the implementation of the policy is the school aged child. Policies often address the needs of school-aged children, since early interventions could help establish positive oral health behaviours, because attitudes towards oral health and oral disease patterns are often established in childhood [1–3].

One of the measures adopted for preventing oral diseases in children in Nigeria is the conduct of school screening exercises, as well as school based oral health education. An example was documented in the report by Sofola et al. [4]. Despite the ethical challenges associated with conducting disease screening exercises [5, 6], the conduct of school screening and school based oral health education programmes in Nigeria has its advantages. One of such is that it helps with the prompt diagnosis and referral of children with oral lesions for management. Past studies in Nigeria [4, 7] showed that these referrals increased dental visits by children, howbeit for curative purposes. Oral health campaigns can reinforce the screening and oral health education processes. It is therefore important that any oral health campaign for children in Nigeria is effective, efficient and designed to achieve results relevant to the Nigerian context.

A national campaign to promote twice-daily tooth brushing among school children in Nigeria was recently introduced because of the need to reduce plaque accumulation and prevent periodontal diseases. This was based largely on the link between periodontal health and heart disease [8, 9]. There are a number of issues raised by this campaign, particularly with regards to its appropriateness for paediatric populations in Nigeria. This paper reviews the oral health status of children in Nigeria, it evaluates the potential impact of the campaign for twice daily brushing, and provides suggestions for how best to address the critical oral health need of children in Nigeria.

**Figure 1:**
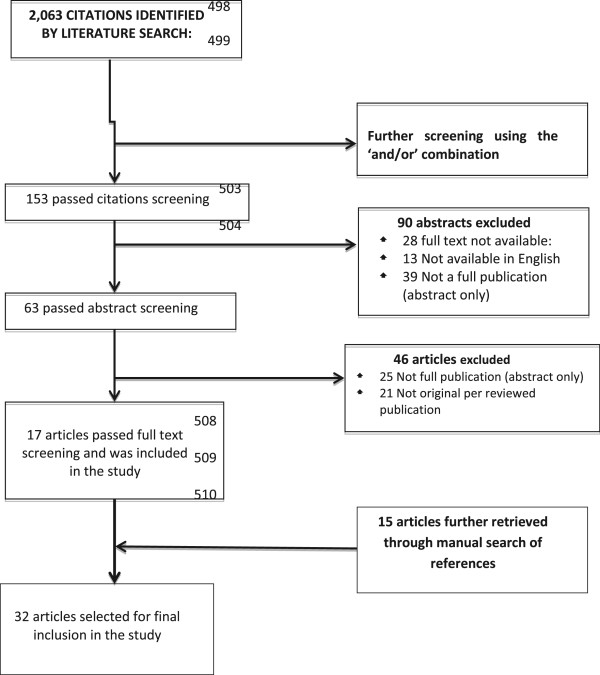
**Literature search flowchart.**

To achieve the objectives of the discussion, a search was conducted for relevant literature using the following data bases; ‘Pubmed’, ‘African Journal online’ and ‘Global health’. The search terms used were ‘dental’, caries’, ‘children’, ‘oral hygiene’, ‘tooth brushing frequency’ and ‘Nigeria’. Initial search retrieved over 2,063 articles, using the ‘and’/‘or’ option reduced it to 153 articles. Review of the retrieved abstracts showed some articles were not relevant while there was some duplication. A total of 63 articles were downloaded, 17 of which were considered relevant for the present study. Where appropriate, the ‘related articles’ search tool was used to retrieve more relevant materials. In addition, the reference lists of all documents and articles retrieved in the previous search strategies were checked, to identify relevant materials. This retrieved a further 15 papers not earlier included. Figure 1 shows the literature search flowchart. Table 1 also gives details of the 32 articles retrieved for this review.

**Table 1:** **Articles discussing caries epidemiology in children in Nigeria**

S. no	Author [reference]	Target population	Age of children	Theme of the study
1	Sofola et al. [4]	In-school	2 years - 12 years	Caries prevalence
2	Folayan et al. [7]	In-school	9 years – 12 years	Oral health service utilisation
3	Akpata [10]	NA	NA	Oral health status
4	Adebola et al. [11]	Hospital based	≤15 years	Oral manifestation of HIV infection
5	Oziegbe and Ezan [12]	In-school	4 years – 16 years	Complication of caries in School children in Nigeria
6	Folayan et al. [13]	In-school	2 years - 12 years	Caries incidence in primary school children
7	Chukumah et al. [14]	Hospital based	≤16 years	Tooth loss
8	Folayan et al. [15]	Hospital based	≤16 years	Tooth loss
9	Ashiwaju et al. [16]	Hospital based	≤16 years	Tooth loss
10	Odia et al. [17]	Hospital based	≤16 years	Reasons for tooth extraction in children
11	Folayan et al. [18]	NA	NA	Caries Epidemiology
12	Sowole and Sote [20]	Preschool	6 months – 5 years	Early childhood caries
13	Abiola et al. [21]	Preschool	18 months – 5 years	Caries and oral hygiene practices
14	Adekoya-Sofowora et al. [22]	Preschool	1-5years	Rampant caries prevalence
15	Umesi-Koleosho et al. [23]	In-school	11 years – 16 years	Caries trend
16	Adeniyi et al. [24]	In-school	5 years – 16 years	Caries prevalence and pattern
17	Okoye and Eknweme [25]	In-school	11 years – 16 years	Caries prevalence
18	Folayan et al. [26]	Hospital based	≤16 years	Risk factors for caries
19	Udoye et al. [27]	In-school	12 years – 15 years	Caries prevalence and pattern
20	Adekoya-Sofowora et al. [28]	In-school	12 years	Caries prevalence
21	Giwa [29]	In-school	12 years	Oral health status of children
22	Folayan et al. [30]	Preschool	6 months – 5 years	Caries risk factor
23	Folayan et al. [31]	Hospital based	1 year – 16 years	Caries risk factor for children
24	Folayan et al. [32]	In-school	8 years – 16 yeas	Preventive oral health practices
25	Sowole et al. [34]	Preschool	6 months – 5 years	Dental caries and oral hygiene practices
26	Folayan et al. [35]	Preschool	6 months – 5 years	Caries and infant feeding practice
27	Adeniyi et al. [36]	Preschool	18 months – 5 years	Oral health risk factors
28	Esan et al. [38]	In-school	2 and 20 years. Mean age: 9.5 yrs ±2.4 yrs.	Oral health status of primary school children
29	Bamgboye and Akande [39]	In-school	11-19 years	Oral health status of secondary school children
30	Kolawole et al. [33]	In-school	Mean age: 12.63 ± 1.06 years	Oral hygiene status
31	Ageblusi and Jeboda [40]	In-school	12 year olds	Oral health status of 12 year old children
32	Okolo et al. [45]	Community based	1-7 years	Oral hygiene and nutritional status

Materials included in this review comprise of articles that addressed the objectives of the study, including the studies that reported on the prevalence and severity of dental caries, and those that reported on dental service utilization. In order to ensure the validity and reliability of the information obtained, we examined the information for consistency and whenever possible, verified it by triangulating it with data in other documents. Information that could not be fully substantiated was excluded.

## Discussion

A variety of dental conditions affect children in Nigeria. They include dental caries [10], periodontal diseases including acute necrotising ulcerative gingivitis [10], traumatic dental injuries [10] and HIV related dental problems [11]. However, dental caries is actually the main oral health concern in children in Nigeria and a problem that requires priority attention. This is because it is associated with significant morbidity [12–17], and negatively influences the quality of life in children.

### The caries burden and profile of children in Nigeria

The prevalence of caries in children in Nigeria is lower than what is observed in many developing countries. The prevalence ranges from between 11.2% and 48.0%: higher in urban than in rural areas, higher in Northern than in Southern Nigeria, and higher in primary than in permanent dentition [18]. The higher caries prevalence in Northern Nigeria has been attributed to the higher cariogenic diet and low manpower to address the oral health needs, relative to what obtains in Southern Nigeria [19]. The prevalence of early childhood caries (ECC) is one of the lowest globally, with prevalence ranging between 5.5% and 10.9% [20–22].

Ethnic differences in caries profile have also been reported [23, 24]. The severity of caries is also comparatively lower than what is observed in many developing countries around the world, with the DMFT ranging between 0.02 and 0.85 in the permanent dentition, and dmft higher than 1.0 only reported in the primary dentition [18]. Caries incidence of 9.9 per 100 children has also been reported, with the relative risk of developing new caries significantly higher in those who already have it [13]. Unfortunately, the treatment of caries is low, with the proportion of children with untreated caries ranging between 77.2% and 98.6% in various communities in Nigeria [18].

Caries prevalence does not differ by sex [25] though it increases with age [26, 27]. The association between socio-economic status (SES) and caries prevalence is less clear, with a study showing that caries prevalence is higher in children from low SES [28], while others show there is no significant difference in caries prevalence by SES [21, 29]. Folayan et al. [30] however showed that the odds of preschool children having caries increased by 23%, as the socioeconomic status decreased.

Children from low socio-economic status however utilise oral health care services less than children from high and middle socio-economic status [31]. A child’s birth rank is not associated with caries risk [31]. However, having older siblings with caries increases the risk of a child having caries [31].

The use of fluoride containing toothpaste in Southern Nigeria is also widespread [32, 33]. Less than a third of school aged children consumed sugar less than once a day [32]. Only 7.8% of school aged children use a combination of caries risk prevention tools (consumption of refined carbohydrate once a day, continuous use of fluoridated toothpaste once a day or more, and restricted intake of refined carbohydrate) [32]. More older children and females use a combination of caries risk prevention tools [32]. The risk of caries increases with the frequency of daily consumption of sugar and with every score increase in oral hygiene index [25, 34]; it also decreases with use of fluoridated toothpaste [24].

Other identified factors associated with the risk of caries among children in Nigeria include; infant feeding practices (children breastfed for more than 2 years have an increased risk for EEC) [35], maternal age (for every year increase in a mother’s age, the odds of the child developing caries increases by one fold) [36] and maternal attitude to oral health (the child’s risk of having caries is reduced by 15% with a unit increase in positive attitude of the mother) [34]. The residential location of the child also influences the form of caries risk the child is exposed to (children from urban areas area more likely to be breast fed on demand and at bedtime, while children from the rural area were more likely to be breast fed on schedule) [29].

Overall, the prevalence and severity of dental caries in children in Nigeria is lower than the 2000 World Health Organisation (WHO) reference standard. Untreated decayed teeth seem to be the main oral health concern in Nigerian children. The second concern is the higher prevalence of untreated dental caries and the relatively higher severity of caries in the primary dentition [4]. Third, risk factors for caries in the primary dentition include maternal factors that if otherwise modified, can improve the oral health behaviour of children.

### Oral hygiene and caries risk in children in Nigeria

Mathiesen et al. [37] showed that there were significantly fewer carious lesions and filled aproximal surfaces in Norwegian children with good oral hygiene compared with children with poor oral hygiene. The results of studies conducted in Nigeria equally corroborate this finding [19, 38]. While the oral hygiene status has been shown to be an important correlate for caries occurrence, no study conducted in Nigeria till date has been able to demonstrate a relationship between frequency of tooth brushing and caries prevalence [38–40]. There were non-statistical differences in the oral hygiene scores of children who brush once a day compared with those who brush twice a day [33]. The importance of tooth brushing efficacy over tooth brushing frequency in reducing the risk for caries had been earlier highlighted [41, 42]. Honkala et al. [41] however noted that the amount of time spent brushing is more closely related to effectiveness of plaque removal than frequency of tooth brushing. Studies show that the practice of twice daily tooth brushing [32, 39] and the use of dental floss by pupils in Nigeria are low [32].

Evidence to support the association between caries prevalence and severity, and tooth brushing frequency is poor [42]. There is also no significant difference in the caries profile of children in Nigeria who brush once a day and those who brush more than once a day [24]. There is therefore no justification for a campaign promoting twice daily brushing as a caries prevention strategy for children in Nigeria.

However the increased frequency of tooth brushing increases the availability of fluoride in the mouth [42]. There are global evidences to demonstrate that fluoride reduces the risk for caries [43, 44]. The risk for caries had also been demonstrated to be reduced in children in Nigeria, who use fluoridated toothpaste [24]. Thus, where and when feasible, campaigns should promote the use of fluoride containing toothpaste by children in Nigeria. Twice daily tooth brushing should be a mechanism to promote increased availability of fluoride in the oral cavity. The promotion of twice daily tooth brushing habits in children also helps to promote the establishment of a habit that helps prevent the development of periodontal diseases [42] in later years.

### Is there any relationship between oral hygiene and periodontal disease in children

In Nigeria, research reports indicate that although oral cleanliness in children is suboptimal with majority of adolescents having fair oral hygiene [40, 45], the prevalence of gingivitis is however, low [40]. Clearly, gingival inflammation is a necessary but not sufficient pre-requisite for periodontitis. Periodontitis is often a manifestation of systemic disease when it occurs in children. Chronic periodontitis has its incipient beginning in adolescence, but is more prevalent among adults. For the most part, surveys indicated that loss of periodontal attachment and supporting bone is relatively uncommon in the young. The incidence is however higher in adolescents aged 12 to 17 years, when compared to children aged five to 11 years [46–49].

The low prevalence of periodontitis in children is due to a number of known factors. First, there is greater anabolic than catabolic activity in children [50] thus making the periodontium more resistant to breakdown. In the unlikely event of breakdown, anabolic activity may enhance concurrent repair. Second, the oral flora is different in children. The aetiological agents of periodontitis – spirochaetes, bacteriodes melaninogenicus and *Porphyromonas gingivalis* – are established late in the oral flora of children [51]. Third, plaque in children has lower irritation potential; oral hygiene can be withheld in preschool children for 27 days with no consequences unlike in adults where inflammatory changes become obvious by the 3^rd^ day [52]. For children in whom the risk for periodontal diseases is low, conducting a major public health campaign to promote twice daily tooth brushing in a bid to reduce periodontal disease in children would be considered inefficient use of resources [53].

### Relationship between severe periodontal disease and heart disease

Evidences suggest that dental health, in particular periodontal disease, may be a significant risk factor for coronary heart disease and further coronary events [54]. The INVEST study however demonstrated equivocally the link between periodontitis and carotid artery plague [55]. The study noted that tooth loss was a marker of past periodontal disease in the study population, and was related to subclinical atherosclerosis thereby providing a potential pathway for a relationship with clinical events. There was however no conclusive causal inferential relationship between any specific heart condition and periodontitis. Rather, there was evidence to suggest increased risk of coronary heart diseases arising from severe periodontal diseases in adults above 40 years of age, particularly men. The American Heart Association also published a report stating that there is no evidence that periodontal disease causes cardiovascular disease, nor is there proof that taking care of your gums will prevent heart disease [56].

Advanced forms of periodontal disease are rare in children though nearly all children and adolescents have gingivitis [47]. The potential risk of coronary heart disease, if there truly is one, is therefore not a concern in childhood. While it may be argued that good habits need to be formed in childhood, there is currently little evidence to substantiate that twice daily tooth brushing significantly reduces plaque formation in children in Nigeria, and therefore, the risk for coronary heart disease that may arise from cumulative events from childhood. The international recommendation for oral health prevention and prophylaxis is daily tooth brushing, with affordable fluoride toothpaste [57].

### What is the evidence for a responsive oral health campaign among children in Nigeria

While the comparatively low caries prevalence and severity in children in Nigeria is impressive, emerging evidence shows that despite school based oral health education programmes, caries incidence in the primary dentition continued to increase, while it decreased in the permanent dentition [4]. Oral health campaigns and programmes on children need to focus on how to reduce caries incidence in the primary dentition, while consolidating the gains made with the permanent dentition. The cumulative gain observed with the National data showing a general decrease in caries prevalence in the country over the last two decades [18], may have actually resulted from a camouflage produced by the decreasing caries incidence in the permanent dentition.

Little is however known or understood about ‘why and ‘how’ the prevalence and severity of dental caries in the permanent dentition had remained low. The widespread public access to fluoridated toothpaste may have played a role in this observed phenomenon [32]. Regular exposure to fluoride through the use of dentifrices when brushing the teeth, reduces the risk of caries [42, 58] *.* The reason for high caries prevalence in the primary dentition in Nigerian children remains unclear. One possibility is that tooth cleansing and the use of fluoridated toothpastes start late in these children. The efficacy of the use of fluoridated toothpaste in the reduction of caries in primary dentition has only been established in a single clinical study [59], while other reviews substantiate the finding [44, 60]. It is important to generate the needed scientific evidence to establish how the gains with caries control in Nigeria were achieved, and identify the challenges faced with caries control in the primary dentition. This will help with the formulation of evidence-based caries control policies and programmes in Nigeria.

What remains clear however is that the number of untreated caries is unacceptably high, suggesting the existence of barriers to utilisation of dental care for children in Nigeria. Efforts need to be directed at addressing this unmet need. There are several publications in Nigerian literature on oral health promotion in children using the school visit approach [23, 26, 61–63]. School screening programmes and referral to dental clinics or hospitals for appropriate care, seems not to have been highly successful in improving the uptake of caries management services [4, 7].

### What should be the focus of the caries management programme for children in Nigeria?

Anecdotal evidence suggests that many of these children do not report to the clinic for treatment, following referrals from school health programmes. It may therefore be more beneficial to include caries treatment strategies in school-based programmes for Nigerian children. Such school based programmes should explore the feasibility of including atraumatic tooth restorative programmes as components of school oral health programmes. Atraumatic tooth restorations have been used successfully in school based programmes in other parts of the world [64, 65]. The introduction of atraumatic tooth restoration into school based oral health programmes would need to be piloted before being scaled up, so that logistic, financial and administrative challenges to programme implementation can be addressed and resolved, prior to nationwide implementation. The National Oral Health Policy [66] has highlighted the importance and role of atraumatic tooth restoration in caries management. Some of the cost associated with the implementation of a school based caries management programme could however be absorbed by the National Health Insurance Scheme (NHIS). The NHIS presently subsidises costs for restorative treatment, but does not subsidise costs for scaling and polishing [67].

A national caries prevention programme that is completely dependent on school based oral health programmes would however leave out a substantial number of children. First, only 60% of primary school aged children are actually in school [68]. Second, a majority of preschool children, who otherwise may be affected by early childhood caries, would not have been enrolled in schools, and would therefore not be reached by the caries prevention programme. In view of this limitation with school based programmes, Nigeria may need to complement her school based oral health programmes for children with programmes that give access to children resident within communities. Community based oral health programmes have helped increase the access of children in underserved communities, to oral health care programmes [69].

Low dental service utilisation may also arise from social and cultural norms. Poor hospital attendance is associated with cultural norms that associate hospital visits with only ill health. This cultural perception may have also deterred people from utilising hospital based oral health services, instead they prefer visiting chemists for dental care services [70, 71]. Also, there are socioeconomic challenges associated with dental service utilisation: pupils living with single mothers or who had no parents, were less likely to utilise dental clinics [72]. Therefore, efforts may need to be focused on addressing those structural problems that impede access of children to dental care services. Access to dental care service is important, as professional tooth cleaning at least once every year, may inhibit caries on all tooth surfaces. Self-performed oral hygiene effectively prevents mainly free smooth surface caries and caries on the front teeth [73].

Caries treatment programmes still need to be complemented with public education programmes. Public education programmes need to emphasise caries prevention, by promoting effective tooth brushing using fluoride containing toothpaste. What we propose is a change in the messages provided on oral health care for children, during public oral health campaigns and education, in Nigeria. Messages for children should facilitate the utilisation of dental care services, for both preventive and prompt management of oral diseases, and promote the use of fluoride containing dentifrices for caries prevention.

## Summary

Allocation of public health resources should be based, where feasible, on objective assessment of health status, burden of disease, their preventability and related cost [74]. This ensures efficient use of the limited financial and human resources. We however do not have enough evidence to suggest that twice daily brushing *per se,* would make any significant impact on caries prevention for children in Nigeria. Twice daily tooth brushing using fluoridated toothpastes would, however, facilitate increased exposure of the oral cavity to fluoride, and make an impact on caries prevention. Mobilising children with caries to receive dental treatment remains the current challenge, and a major oral health need that should be addressed. Children focused oral health campaigns in Nigeria should therefore promote improved uptake of dental services, and access to dental care providers.
